# Performance output data and configurations of stencil compilers experiments run through PROVA!

**DOI:** 10.1016/j.dib.2018.08.092

**Published:** 2018-08-30

**Authors:** Danilo Guerrera, Antonio Maffia, Helmar Burkhart

**Affiliations:** University of Basel, Switzerland

## Abstract

The data in this article are related to the research article titled “Reproducible Stencil Compiler Benchmarks Using PROVA!”. Stencil kernels have been implemented using a naïve OpenMP (OpenMP Architecture Review Board, 2016) [1] parallelization and then using the stencil compilers PATUS (Christen et al., 2011) [2] and (Bondhugula et al., 2008) PLUTO [3]. Performance experiments have been run on different architectures, by using PROVA! (Guerrera et al., 2017) [4], a distributed workflow and system management tool to conduct reproducible research in computational sciences. Information like version of the compiler, compilation flags, configurations, experiment parameters and raw results are fundamental contextual information for the reproducibility of an experiment. All this information is automatically stored by PROVA! and, for the experiments presented in this paper, are available at https://github.com/sguera/FGCS17.

**Specifications Table**

**Table:** 

Subject area	*Computer Science*
More specific subject area	*High Performance Computing*
Type of data	*Shell scripts, JSON files (descriptors, performance output data)*
How data was acquired	*Execution of the compiled source code*
Data format	*Raw*
Experimental factors	
Experimental features	*Several kernels with different properties: grid size, number of threads used in the parallel execution. Two architectures (compute systems). Three implementation approaches: naïve openMP, and 2 stencil compilers: PATUS and PLUTO*
Data source location	*Basel (Switzerland) and Erlangen (Germany)*
Data accessibility	https://github.com/sguera/FGCS17.
Related research article	Reproducible Stencil Compiler Benchmarks Using PROVA!

**Value of the data**

• The data are output of reproducible executions of the source codes on different architectures, from CPU to GPU.
• The data allow to compare the performance obtained using different approaches to parallelize stencil computations: naïve OpenMP (OpenMP Architecture Review Board, 2016) [1], PLUTO (Bondhugula et al., 2008) [3] and PATUS (Christen et al., 2011) [2].compilers).
• Several stencils have been implemented and their execution performance registered, thus broadening the impact of these data to several communities.
• The configuration data available, allow to reproduce the experiments eve n without using PROVA! (Guerrera et al., 2017) [4] (a tool for reproducible research).

## 1 Data

Information like version of the compiler, compilation flags, configurations, experiment parameters and raw results are fundamental contextual information for the reproducibility of an experiment. All these information are automatically stored by PROVA! when creating a project, a method, or an experiment. Each of them holds a descriptor that stores the relevant information and makes them available to the tool and the users, when needed.

For each used software PROVA! stores the building and installing recipes, in the form of easyconfig files (used by EasyBuild), the compilation and execution commands, together with their environment (automatically using environment modules, in a way transparent to the user), self-documenting the whole research from the creation of a project until the run of an experiment.

Thus the general structure of the data in the repository is:

• PROBLEM
  ○ EASYCONFIGS
  ○ COMPUTE SYSTEM
    ▪ METHOD (implementation)
      ● OUT (raw output per thread used)
      ● SRC
        ○ Makefile
        ○ Sources
        ○ INPUT_PARAMETERS
      ● METHOD DESCRIPTOR
    ▪ EXPERIMENTAL RESULTS
      ● PROLOGUE infos
      ● EPILOGUE infos
      ● RESULTS (ordered in a json)
      ● PLOTTING SCRIPT
      ● FIGURE
    ▪ METHOD_TYPE
      ● COMPILATION SCRIPT
      ● RUN SCRIPT
      ● METHOD_DESCRIPTOR (containing the environment module used)

## 2 Experimental design, materials, and methods

### 2.1 Experiment 1

In this experiment has been solved a classical wave equation with a fourth order-in-space and second order in time finite difference method. After the discretization, 3 implementations have been produced:

• a naïve C + OpenMP
• a C source with PLUTO directives
• a source in a Domain Specific Language used as input for PATUS.

Two different compute systems have been used:

• Mint cluster at university of Basel: 4 compute nodes of dual socket AMD Opteron 6274 “Bulldozer” with a nominal clock speed of 2.2 GHz and 16 cores per chip. Each CPU has a 2 × 6 MiB L3 cache shared among a NUMA domain (8 cores), every 2 cores are sharing a 2 MiB L2 cache (16 MiB in total), and a 16 KiB core private L1 cache. The peak performance of one core is 8 flops per cycle in DP or 16 flops per cycle in SP. Each node is equipped with 128 GiB of RAM (DDR3–1600) per socket and has a maximum memory bandwidth of circa 20 GB/s per socket (measured via the STREAM COPY benchmark). The machine is running a 64-bit Ubuntu 14.04.4 LTS (kernel 3.13.0-93-generic).
• LiMa cluster at Regionales RechenZentrum Erlangen: 500 compute nodes of dual socket Xeon 5650 “Westmere” with a nominal clock of 2.66 GHz and 6 cores + SMT per chip. Each CPU has 12 MiB L3 cache shared among all cores, and core-private L2 and L1 caches of 256 KiB and 32 KiB respectively. The peak performance of one core is 4 flops per cycle in DP or 8 flops per cycle in SP. Each node is equipped with 24 GiB of RAM (DDR3-1333) per socket and has a maximum memory bandwidth of circa 21 GB/s per socket (measured via the STREAM COPY benchmark). The machine is running a 64-bit *CentOS* release 6.7(kernel 3.10.0-327.22.2.el7.x86_64).

The software stack, maintained by PROVA!, consisted, respectively, of the following environment modules:

• GCC/4.9.3-2.25
• GCC/4.9.3-2.25, PLUTO-pet/0.11.0 (pet branch)
• GCC/4.9.3-2.25, PATUS/0.1.4, Java/1.7.0_79, Maxima/5.37.2 (compiled with ecl/16.0.0)

The easyconfigs used to install such modules are available at the repository.

The experiment has been conducted using three dimensional grids of size 200^3^ and *IEEE* single precision arithmetic (float), over 100 timesteps.

### 2.2 Experiment 2

The second problem we chose to solve is a blur filtering. The filter matrix of the smoothing used, corresponds to a discrete two dimensional Gaussian function: $G\left(x,y\right)=\frac{1}{\sqrt{2\pi \sigma^{2}}}\exp(-\frac{x^{2}+y^{2}}{2\sigma^{2}})$, where *σ* denotes the width (i.e. standard deviation) of the bell-shaped function. Gaussian filters are isotropic if the filter matrix is large enough (at least 5 × 5, like in our case) to provide a sufficient approximation.

The size of our grids is 1024^2^ points and we calculate 50 *timesteps* in *IEEE* single precision arithmetic.

The same systems and methods of Experiment 1 have been used.

### 2.3 Experiment 3

A classical heat equation, describing the temperature change over time, given initial temperature distribution and boundary conditions. A finite differencing scheme is employed to solve the heat equation numerically on a square region. The size of the grids is 512^2^ and we calculate 100 timesteps in IEEE single precision arithmetic.

The implementation used makes use of MPI for the parallelization.

The compute system used is Mint (details presented above). To complement that description, it is equipped with Mellanox Infiniband MT26428 [ConnectX VPI PCIe 2.0 5GT/s - IB QDR / 10GigE].

The module used is OpenMPI/1.10.1-GCC-4.9.3-2.25.

### 2.4 Experiment 4

The problem solved is the same presented in the Experiment 1, using a naïve implementation, parallelized with OpenMP, using NUMA-aware initialization. The compute system where it was run is the KNL partition of the

• miniHPC cluster: 22 compute nodes of dual socket Xeon E5-2640 v4 “Broadwell” with a nominal clock of 2.4 GHz and 10 cores+SMT per chip. Each CPU has 25 MiB L3 cache shared among all cores, and core-private L2 and L1 caches of 256 KiB and 32 KiB respectively. The peak performance of one core is 16 flops per cycle in DP or 32 flops per cycle in SP. Each node is equipped with 32 GiB of RAM (DDR4-2133 ECC) per socket and has a maximum memory bandwidth of circa 35 GB/s per socket (measured via the STREAM COPY [48,49] benchmark). Additionally, 4 KNL nodes are available, each being a 64 Core Intel Xeon Phi 7210 1.50 GHz Processor (16GB on-Die Memory), equipped with 96GB 2133 MHz DDR4 ECC memory and Omni-Path 100 GBits Host Fabric Interface, with a maximum memory bandwidth of 102 GB/s (as displayed on the Intel website). The machine is running a 64-bit CentOS release 7.2.1511(kernel 3.10.0-327.el7.x86_64).

The environment module used is GCC/ 4.9.3-2.25.

### 2.5 Experiment 5

In this experiment a 2D Jacobi calculation is carried out, computing a new value for each element of the grid, such a value being an average of the element׳s current value and the values of its neighbors. Several grid sizes were used (512^2^, 1024^2^, 2048^2^ and 4096^2^), calculating 10,000 time-steps. The implementation uses CUDA to parallelize the kernel. The experiments ran on a machine hosting a

• NVIDIA GeForce GTX 1060: 10 Multiprocessors, 128 CUDA Cores/MP, warp size of 32, 1.85 GHz max clock rate, 1.5 MiB L2 cache and 6 GiB of 4 GHz RAM. The device to device memory bandwidth is circa 14 GB/s, while the host to device and device to host peaks at 12GB/s (measured via the NVIDIA bandwidth test, available in CUDA).

The environment module used is CUDA-8.0.44-GCC-5.4.0-2.26.
